# Indiscriminate Discharge of Insane

**Published:** 1902-02

**Authors:** 


### Indiscriminate Discharge of Insane.

Apropos of the *Journal's* recent criticisms of the laxity displayed by the managers of insane asylums in permitting dangerous lunatics to have their liberty, often at the cost of one or more sane and innocent lives, is a recent occurrence in a large city not many miles from Cleveland. A paretic, who was at times afflicted with destructive delusions, had not long before become an inmate. In a very short time, under intelligent care and away from sources of irritation, his manner became quite mild, and his family and friends made up their minds that he was cured. They applied for his release, which was very properly refused by the superintendent, whose knowledge and experience enabled him to assert with confidence that the disease with which the patient was afflicted was progressive and incurable, and that freedom would afford opportunity for the recurrence of fits of violence. The family were now sure that the patient was being improperly restrained against his will, and an attorney was appealed to for help in obtaining the coveted discharge. The attorney went to the asylum, had a half-hour interview with the patient, and immediately informed the superintendent that the paretic was as sane as anybody, adding that it was an outrage to confine a sane man among a lot of lunatics. In spite of much sputter the superintendent courageously maintained his ground, and the attorney was compelled to leave without accomplishing his object, but took occasion to make a number of harsh remarks about the treatment of the insane. In a few days he returned, triumphantly laying on the superintendent's desk a letter written by the judge who had committed the patient and who now endorsed the request for his release. The superintendent, knowing the dangerous character of the patient's mental condition, still refused to assume the responsibility of his discharge, and pointed out the fact that no one else had authority to issue the order. The attorney, being of excitable disposition, almost exploded with righteous wrath, and threatened all kinds of legal terrors. He laughed at the assertion that the patient was dangerous. He had "talked with him for half an hour, and he is just as sane and harmless as you or I." Finally the superintendent, realizing the harm that could be done the institution by loudly repeated stories of the restraint of sane people, told the attorney that he could have his client on the one condition that he (the attorney) would execute in legal form a bond assuming all responsibility for the patient's acts after his discharge. After some grumbling the bargain was made, and the next morning the attorney appeared with his bond. He then took the patient away with him, remarking that he was proud to have been able to secure the release of one unfortunate from such a brutally managed institution!

On the evening of the same day the superintendent was called to the telephone and informed by the police that they had the patient at the station. They had been called by the family because the patient after a few hours had gone to work breaking up furniture, attempting to fire the house, and threatening violence to everything and everybody. In their terror at the actions of their "sane" relative the family appealed for police protection! The police asked what they should do with the prisoner. The superintendent, with perfect equanimity, replied: "Mr. X., an attorney-at-law, has assumed entire charge of that patient. Report to him." —*Cleveland Journal of Medicine.*

